# Agronomic and Environmental Assessment of a Polyculture Rooftop Soilless Urban Home Garden in a Mediterranean City

**DOI:** 10.3389/fpls.2019.00341

**Published:** 2019-03-22

**Authors:** Anna Boneta, Martí Rufí-Salís, Mireia Ercilla-Montserrat, Xavier Gabarrell, Joan Rieradevall

**Affiliations:** ^1^Sostenipra Research Group (2017 SGR 1683), Institut de Ciència i Tecnologia Ambientals (ICTA-UAB), Universitat Autònoma de Barcelona, Barcelona, Spain; ^2^Department of Chemical, Biological and Environmental Engineering, Universitat Autònoma de Barcelona, Barcelona, Spain

**Keywords:** urban agriculture, soilless, polyculture, rooftop farming, home garden, life cycle assessment

## Abstract

Urban planning has been focusing its attention on urban rooftop agriculture as an innovative way to produce local and reliable food in unused spaces in cities. However, there is a lack of quantitative data on soilless urban home gardens and their contribution to self-sufficiency. The aim of the present study is to provide quantitative agronomic and environmental data on an actual soilless urban garden to estimate its degree of self-sufficiency and sustainability. For this purpose, an 18 m^2^ soilless polyculture rooftop urban home garden in the city center of Barcelona was analyzed. From 2015 to 2017, 22 different crops were grown to feed 2 people in an open-air soilless system, and a life cycle assessment was performed. A total productivity of 10.6 kg/m^2^/year was achieved, meaning that 5.3 m^2^ would be needed to fulfill the yearly vegetable requirements of an average citizen (in terms of weight). Considering the vegetable market basket of Catalonia, an 8.2 m^2^ soilless garden would be needed to cover 62% of the market basket for one person. The top 5 most productive crops were tomato, chard, lettuce, pepper and eggplant, accounting for 85.5% of the total production. The water consumption was 3.7 L/m^2^/day, and 3.3 kg/year/m^2^ of waste was generated. A high degree of self-sufficiency was achieved, although adjustments could be made to adapt the production to the market basket. The environmental assessment showed that the fertilizers and their associated leachates accounted for the highest environmental impacts in all the studied impact categories. Overall, 0.6 kg CO_2_ eq. was generated per kg of vegetables produced. The quantitative data provided by the present study offer a reference from which urban planners and researchers can project future implementations of rooftop urban agriculture (UA) on a large scale.

## Introduction

The continuously increasing world population is predicted to rise to almost 10 billion people by 2050 (United Nations, Department of Economic and Social Affairs, Population Division, 2017). This situation will lead to a higher food demand and, consequently, increased pressure on many ecosystem services (Watson et al., 2011). Moreover, the population living in urban areas is also expected to increase from 54% (in 2015) to 66% by 2050 (WHO, 2016). Nevertheless, in the European Union, 72% of the population already lives in urban areas (Eurostat, 2015). The consequence of this trend is a progressive urban expansion that widens the distances between production and consumption areas, increasing the dependence of cities on external resources (Scoones, 2009). In this context, the necessity of rethinking our food systems is rising to achieve urban sustainability and avoid intensive agricultural techniques that have critical environmental costs.

As a result, urban agriculture (UA) is gaining importance to facilitate access to healthy, reliable and fresh food, which is usually difficult in cities (e.g., “food deserts”) (WHO, 2016) along with many other social and ecological related services. Specifically, urban rooftop farming, which includes gardens, greenhouses or farms placed on building rooftops, can offer new landscape opportunities while restraining the burden on agricultural land and achieving more sustainable and resilient cities (Thomaier et al., 2015). Such is the case in which urban planners in northern global cities already include UA in their agendas and policy planning (Morgan, 2009).

Although soil-based agriculture is the most common urban agricultural practice (Thomaier et al., 2015), soilless systems are gaining importance as the lightest operation system. Therefore, UA can be performed in unused urban spaces, such as rooftops or terraces (Nowak, 2004), which are already built spaces that are usually empty (Thomaier et al., 2015). Moreover, one of the major risks in UA is contamination, mainly caused by heavy metals present in soils (Ercilla-Montserrat et al., 2018). In this sense, soilless practices help avoid this risk by using inert and non-contaminated substrates (Ercilla-Montserrat et al., 2018). Notwithstanding that soilless systems can be perceived as “unnatural” or artificial (Specht and Sanyé-Mengual, 2017), it should be considered that this practice is already highly consolidated in conventional agriculture (Savvas et al., 2013). For instance, intensive greenhouse soilless food production is performed in Almeria (Spain), the major vegetable producer in southern Europe. According to Specht and Sanyé-Mengual (2017), many of the vegetables for sale in the market are already produced using soilless techniques (Specht and Sanyé-Mengual, 2017). This wide use of soilless systems is due to the substantial water savings that it allows (Thomaier et al., 2015). Although irrigation management is crucial for the performance of soilless systems, easy access to nutrients and water allows plants to grow faster and produce higher yields at higher densities because there is no competition for nutrients (Nowak, 2004).

Apart from community, commercial or industrial UA initiatives, private home gardens have been always present and still discreetly sprouting in cities (Kortright and Wakefield, 2011). According to Calvet-Mir et al. (2012), there are many reasons to cultivate home gardens. The main goal is to obtain better quality and safer food, which will consequently enhance healthier diets by increasing the intake of abundant and diverse vegetables (Grewal and Grewal, 2012). Another important reason is that home gardens increase self-reliance and self-sufficiency, allowing certain economic independence and resilience to external dynamics (Calvet-Mir et al., 2012). Therefore, food sovereignty can be seen as a form of empowerment (Grewal and Grewal, 2012). In terms of production, Sanyé-Mengual et al. (2013) quantified that 150 tones of tomatoes could be produced in the roof area of Barcelona. Although concerns about community and industrial UA are gaining interest worldwide, home urban gardens are overlooked and understudied (Taylor and Lovell, 2014). In addition, the existing literature concerning home urban gardens is mainly qualitative and focused on their ecosystem and social services provisions (Calvet-Mir et al., 2012; Cameron et al., 2012) or their contribution to food security (Kortright and Wakefield, 2011) rather than on their agronomic and environmental performance.

From an environmental impact perspective, urban food production has been assessed for rooftop greenhouses (Sanjuan-Delmás et al., 2018) and community rooftop gardens (Sanyé-Mengual et al., 2015) by applying the Life Cycle Assessment methodology (ISO 14014). Nevertheless, there is still a gap in the literature regarding agronomic and environmental studies on open-air, urban, soilless and polyculture gardens. As stated by Specht and Sanyé-Mengual (2017), the available literature is insufficient, and new quantitative data are needed to “increase awareness and knowledge” about urban rooftop agriculture.

The present research seeks to address this gap in the literature by performing a quantitative analysis of the agronomic and environmental performance of a soilless urban home garden in a Mediterranean city that grows a wide variety of vegetables (i.e., polyculture) using life cycle tools. In addition, this paper will provide useful specific indicators for policy making and design planning in cities that seek to enhance UA. There is little information in the literature about open-air rooftop soilless polyculture home urban gardens, and assessing the potential yield for larger-scale urban production could be useful. This lack of data implies uncertainty in urban management and hinders the inclusion of a food policy dimension in urban plans. This study seeks to shed some light by providing new quantitative data to assure the best performance of urban gardens in the future.

The general aim of this paper is to assess the feasibility of an open-air rooftop soilless polyculture home urban garden in Barcelona by providing quantitative agronomic and environmental data. Two specific goals were designed, which are related to the main section of the present study:

• To assess the agronomic performance of the system and relate it with the food demand of the citizens to assess the amount of area needed to fulfill the vegetable consumption of the city.
• To assess the environmental impacts of a real case study by applying the life cycle assessment methodology.

## Materials and Methods

In this section, detailed information can be found on the system under study as well as the experimental design and the agronomic and sample collection. Additionally, the stages of the life cycle assessment methodology applied for the environmental impact assessment are presented.

### Study Area

The garden under study is 18 m^2^, and it is placed on a rooftop (private terrace on the first floor above the street level, south-facing) in the city center of Barcelona (41.38481N, 2.163125E). Barcelona is a very dense city, with almost 16,000 inhabitants per km^2^ (IDESCAT, 2018) due to its geographic location, which impedes its sprawl. The scarce land availability triggers urban rooftop agriculture as a potential solution for many urban sustainability issues related to the food supply. Moreover, the Mediterranean climate allows year-round open-air farming practices. The general meteorological data for the years under assessment are presented in Table 1. As can be observed, there are no major temperature changes across years. However, the rainfall was higher and more densely concentrated in fewer days in 2017 than in previous years.

**Table 1:** General meteorological data in the city of Barcelona (Ajuntament de Barcelona, 2018a).

		2015	2016	2017
Temperature	Average (°C)^1^	18.4	18.4	18.4
	Maximum (°C)^1^	34.7	32.6	34.2
	Minimum (°C)^1^	2.1	3.9	1.6
Rainfall	Total (mm)^1^	316.9	438.5	478.9
	Maximum in a day (mm)^2^	47.0	66.0	115.1
	Rainy days^2^	95	119	109

^1^*Can Bruixa Observatory. ^*2*^Fabra Observatory*.

### Experimental Design

The years under assessment are 2015, 2016, and 2017. Data on the different inputs and life cycle stages are detailed in Supplementary Information 1. Moreover, to add data reliability, 2014 was excluded from the analysis because it was mainly used to build and adjust the whole system.

**Figure 1:**
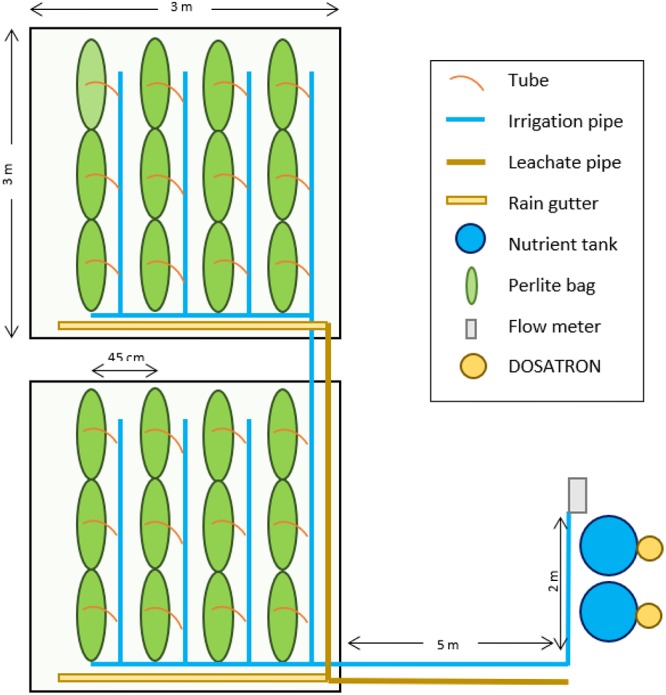
Scheme of the system under study.

A soilless operation system was used in which plants grow in an inert substrate (perlite) and nutrients and water are supplied through fertigation. As Figure 1 shows, 24 perlite bags were installed, and there was room for 72 plants (3 per bag). Each bag contained 40 L of perlite and measured 1 × 0.3 m. Distance between bags (45 cm) and between crops is necessary to perform management tasks and to allow the cultivation of both fruit and leaf crops. As different crops were growing simultaneously, the plant density remained constant for the whole period (4 plants m^-2^). Although smaller distances would have allowed higher crop densities, polyculture gardens have to adapt to crop versatility and have to be managed to satisfy the needs of all kinds of crops (e.g., avoid shadowing between plants).

The water came from two polyethylene 50 L tanks that were filled with water from the municipal water network. Nutrients were injected from a concentrated nutrient solution designed by agronomic experts, and the irrigation flow was distributed through drippers. The water system was open (or linear), and thus, the leachates were disposed of. The leachates were not quantified for this study, but data from Sanjuan-Delmás et al. (2018) (based on a hydroponic tomato crop in a nearby area) was extrapolated for their quantification and composition. The nutrient solution (Table 2) was generic for the different crops grown (non-specific for leaf or fruit products).

**Table 2:** Fertilizer irrigation concentration.

					CaCl_2_^∗^
	KPO_4_H_2_	KNO_3_	K_2_SO_4_	Ca(NO_3_)_2_	2H_2_O	Mg(NO_3_)_2_	Hortilon	Sequestrene
mg ⋅ l^-1^	136	101	217.5	164	111	148.3	10	10

### Agronomic Data

**Figure 2:**
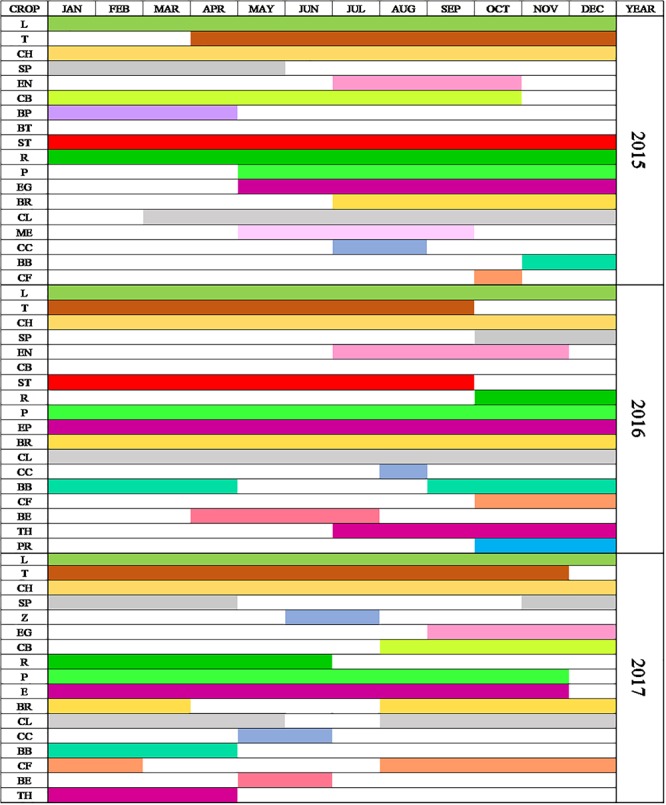
Time data on when the crops occupied a space in the urban garden. Abbreviation meanings: L, Lettuce; T, Tomato; CH, Chard; SP, Spinach; Z, Zucchini; EN, Endive; CB, Cabbage; BP, Green pea; BT, Beetroot; ST, Strawberry; R, Arugula; P, Pepper; EG, Aubergine; BR, Broccoli; CL, Celery; ME, Melon; CC, Cucumber; BB, Broad bean; BE, Bean; TH, Thistle; and PR, Parsley.

Trials were performed for 22 different crops (Table 3), including leafy and fruit vegetables. The study started with 5 pilot crops in 2014 and increased to 19 in 2015 and to 22 in 2016 and 2017. The inclusion criteria for the crops were their feasibility to be grown in soilless systems and open-air conditions and their representability in the Mediterranean diet. Two main different types of crops were grown: fruit and leafy crops. The main difference is that while leaf products are harvested by uprooting the whole crop, fruits have staggered harvests during a longer period. Additionally, leaf crops have shorter cycles and can be planted many times a year, while fruiting vegetables are planted only once or twice a year. Data on when the crops grew are shown in Figure 2. The yield data considered the fresh weight of the plants. This combination and proper management results in minimum variation in the seasonal yield and the efficient use of space. According to Orsini et al. (2014), both are essential in self-sufficient urban home gardens.

**Table 3:** List of crops grown in the present study.

Common name	Scientific name	Variety
Aubergine	*Solanum melongena*	–
Bean	*Phaseolus vulgaris*	Contender
Beetroot	*Beta vulgaris*	Rubra
Broad Bean	*Vicia faba*	Histal
Broccoli	*Brassica oleracea*	Italica
Cabbage	*Brassica oleracea*	Capitata
Cauliflower	*Brassica oleracea*	Botrytis
Celery	*Apium graveolens*	Dulcis
Chard	*Beta vulgaris*	Cicla
Zucchini	*Cucurbita pepo*	–
Cucumber	*Cucumis sativus*	–
Endive	*Cichorium endivia*	Crispum
Green pea	*Pisum sativum*	–
Lettuce	*Lactuca sativa*	Meravella
Melon	*Cucumis melo*	–
Parsley	*Petroselinum crispum*	Apium petroselinum
Green pepper	*Capsicum baccatum*	–
Arugula	*Eruca vesicaria*	–
Spinach	*Spinacia oleracea*	–
Strawberry	*Fragaria vesca*	–
Thistle	*Cynara cardunculus*	
Tomato	*Solanum lycopersicum*	Arawak

Data from the Catalan Administration (Gencat, 2016) on food consumption preferences were gathered to assess how the garden under study could cover the food demand of the citizens.

### Sample Collection

Data were collected manually for each different crop every harvesting day, indicating the weight per plant (for the leafy crops) and the fruit yield per plant for the fruit crops. The data were converted to the yield per square meter considering all of the garden area. Information about irrigation changes (according to the season) and fertilizer reposition was also collected, indicating the date when these activities took place. In addition, details about the overall state of the crops and the system were also acquired. No technical advice (apart from that which was needed to ensure data quality) or improvement suggestions were provided by the researchers during the study period. The management was fully performed by the garden’s owner.

### Life Cycle Assessment

The environmental impacts were quantified using a Life Cycle Assessment following ISO 14040-44 (ISO, 2006), which includes four main stages.

#### Goal and Scope

The LCA included the whole garden system (from raw material extraction to end of life), excluding the nursery plants (considered negligible based on the data from Sanjuan-Delmás et al. (2018). No extra energy was needed except the amount included in the tap water supply from the network. The system was split into infrastructure (elements with a lifespan of more than 5 years) and operation (elements with a lifespan of less than 5 years) (Figure 2).

The functional unit selected for this assessment was 1 kg of edible fruit and leafy products cultivated in an open-air soilless polyculture rooftop home garden in a Mediterranean city. Moreover, the impacts per year were also used in some sections of the discussion to add clarity.

#### Inventory

**Figure 3:**
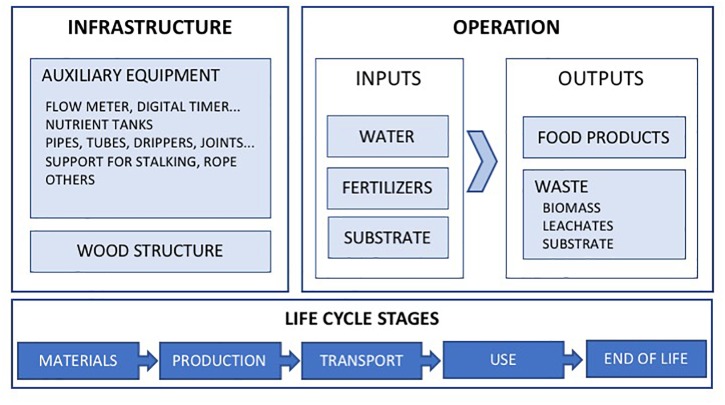
Diagram of the system boundaries.

The Life Cycle Inventory (LCI) is the data collection portion of the LCA. In this section, all the inputs and outputs are described, indicating the data sources used to define the origin, transport and end of life of each element or flow. In addition, the assumptions taken into account for the analysis are presented. The system boundaries are graphically described in Figure 3.

##### Infrastructure

The complete inventory of the auxiliary equipment can be found in Supplementary Information Table A1. It is considered that all the elements are transported with a light commercial vehicle from a distributor 35 km away from Barcelona. For the waste scenario, it is considered that most of the equipment is recycled at a recycling plant 10 km away from Barcelona and transported in a municipal waste collection lorry. The leachate tray (made of expandable polystyrene) was not recyclable and was taken to a landfill 25 km away from the system. Regarding the wood structure, the end of life treatment was the same as that used by Puy et al. (2010) in Scenario 1 (postconsumer wood from the recycling points). This treatment includes the transport of the wooden waste to the nearest recycling point and from there to a treatment plant where the wood is separated and chipped (24 km away). Finally, the product is transported to a gasification plant in Cerdanyola del Vallès (36 km) where energy is generated from the wooden waste.

##### Operation and maintenance

The water used for irrigation came from the municipal water network (tap water), and the potabilization treatment impacts were included in the assessment. Substrate bags were not available locally and therefore imported from Almeria (800 km). With a lifespan of 3 years, the substrate bags are finally taken to a landfill as an inert material. The fertilizers traveled 33 km in a transport van to the system. They were concentrated in a nutrient solution and then injected into the irrigation flow, which was approximately 2 L/h. The fertilizers leave the system as leachates in the sewer network and end up in a wastewater treatment plant. Some of these treatment plants do not remove all the chemicals; thus, the impacts associated with the leachates were accounted for as direct emissions to water (Sanjuan-Delmás et al., 2018). The residual biomass was deposited in a municipal organic waste container and transported to a composting plant 13,5 km away from Barcelona. The complete inventory of the operation phase can be found in Supplementary Information 1 Table A1.

### Life Cycle Impact Assessment

The LCA was performed using Simapro 8.2 software by Pré Consultants. The method used to calculate the environmental impacts was ReCiPe (hierarchical) at a midpoint level. The Ecoinvent 3 database was used as the main source for the background environmental data. According to Brentrup et al. (2004), the selected impact categories for the assessment were the following: climate change (CC), ecotoxicity (ET; which is calculated by adding terrestrial, freshwater and marine ecotoxicity environmental impacts), terrestrial acidification (TA), freshwater eutrophication (FE), marine eutrophication (ME), and fossil fuel depletion (FDP).

## Results

The agronomic and environmental performance of the study system are shown and described in the following section. Food production is assessed for the agronomic portion, while the environmental performance focuses on the life cycle impact assessment considering the data acquired in the inventory.

### Agronomic Data and Food Production

As shown in Table 4, a total of 569.7 kg of vegetables were produced during the study period (2015–2017), of which 354 kg were fruits and 215.7 kg were leafy vegetables. By splitting the data in time, an average productivity of 189.9 kg/year and 10.6 kg/m^2^/year was achieved.

**Table 4:** Agronomic results from 2015, 2016, and 2017 for the present study.

							Total
							production
		Unit	2015	2016	2017	Average	2015–2017
Lettuce	L	Total kg	22.3	27.4	19.6	23.1	69.3
Chard	L		33.5	14.6	32.9	27.0	81.0
Spinach	L		10.5		5.0	7.8	15.5
Tomato	F		54.5	29.8	35.6	40.0	119.9
Zucchini	F				1.0	1.0	1.0
Endive	L		1.5	1.95		1.7	3.5
Cabbage	L		15.7			15.7	15.7
Green pea	F		2.8			2.8	2.8
Strawberry	F		0.1	0.7		0.4	0.8
Arugula	L		0.7		1.6	1.2	2.3
Pepper	F		30.4	25.2	30.3	28.6	85.9
Eggplant	F		15.3	29.7	27.6	24.2	72.6
Broccoli	F		3.4	3.9	10.1	5.8	17.4
Celery	L		4.4	15.5	1.5	7.1	21.4
Melon	F		3.6			3.6	3.6
Cucumber	F		1	1	2.5	1.5	4.5
Broad bean	F			5.7	8.6	7.2	14.3
Cauliflower	F		4		15.0	9.5	19.0
Bean	F			4.5	5.5	5.0	10.0
Thistle	L			3.9	3.2	3.6	7.1
Artichokes	F				2.2	2.2	2.2
Total leafy production			88.6	63.4	63.8	71.9	215.7
Total fruit production			115.1	100.5	138.4	118.0	354.0
Total production			203.7	163.9	202.2	189.9	569.7
Total production		kg/m^2^	11.3	9.1	11.2	10.6	
Waste biomass		kg/m^2^	3.8	2.8	3.3	3.3	
Water		L/(m^2^ ⋅ d)	3.3	3.6	4.2	3.7	
		L/kg	107.7	146.0	137.8	130.5	

*Classification of the leafy (L) and fruit (F) products*.

The top 5 most productive crops were tomato, lettuce, chard, pepper and eggplant (in this order), representing 85.5% of the total production. Specifically, the average top 3 leaf vegetables were chard, lettuce and cabbage, with an average production of 27, 23.2, and 15.7 kg/year, respectively. The 3 most productive fruit crops were tomato, pepper and eggplant, with an average of 40.0, 28.6, and 24.2 kg/year, respectively. Table 5 shows the specific results in these 3 crops from 2015 to 2016.

**Table 5:** Production data for 3 crops in the present study in 2015 and 2016.

		Number of
Crop	Season	plants	DAT	kg	plant/m^2^	kg/plant	kg/m^2^
Tomato	August–March	18	221	16.3	4	0.91	**3.62**
	March–September	12	169	17.5		1.46	**5.83**
Pepper	May–January	6	250	18.9		3.15	**12.60**
	July–March	6	267	16.4		2.73	**10.93**
Eggplant	May–March	3	310	17.1		5.70	**22.80**

*DAT indicates days after transplanting*.

The tomato summer and winter season productions resulted in 5.8 kg/m^2^ and 3.6 kg/m^2^, respectively. Compared with other studies, such as Orsini et al. (2014), who cultivated tomato in an urban rooftop with a soil-based system, or Sanjuan-Delmás et al. (2018), who grew tomato in an integrated rooftop greenhouse with a soilless system, this is a relatively low production. The differences between the yield obtained in the summer and winter crops were mainly caused by meteorological conditions, as the system in this study is open-air. However, the differences in yield between seasons were less different than those obtained outside urban area (Sanjuan-Delmás et al., 2018).

In contrast, the pepper crops produced 12.6 kg/m^2^ during the summer season and 10.9 kg/m^2^ during the winter season. In this case, the production is considerably high, even surpassing the industrial pepper production in soilless greenhouses (García Lozano et al., 2005). Similarly, the eggplant production (22.8 kg/m^2^) was highly significant and exceeded the annual production in Spain (4.5 kg/m^2^) (Daunay, 2008).

The other crops, such as melon and green peas, had very low yields (<5 kg per year) and presented fungal infections. Therefore, they were not cultivated the following years. A few of the crops, such as beetroot or parsley, were not successful and were not considered in the analysis.

As a reference for local conventional production, the annual Catalan production of tomato, pepper and eggplant is 4.10, 2.45, and 2.56 kg/m^2^, respectively (IDESCAT, 2018). These data show that the home garden productivity of these crops was significantly higher, except for the winter tomato crop.

The number of people that the garden could cover was calculated (Supplementary Information 2 Table B1). Considering that the home with the garden under study hosted two people, the consumption was adequately covered by the garden production except for zucchini, bean and cucumber production (10%, 75–90%, and 20–55% for the demand of 2 people). For crops such as chard or eggplant, the production was largely exceeded (487–1116% and 364–707% for the demand of 2 people, respectively).

### Environmental Assessment

In this section, the Life Cycle Impact Assessment (LCIA) is performed by analyzing every subcategory of the inventory. The most important impacts are highlighted, and some recommendations for improvements are given.

#### Life Cycle Inventory

The inventory of the two subsystems considered (infrastructure and operation) is described in detail in Supplementary Information 1 Tables A1, A2.

#### Life Cycle Impact Assessment

Table 6 shows the environmental impacts per kg of product as well as the relative impact per impact category. The results show that most of the environmental impacts are generated during the operation phase of the garden (water, substrate, waste biomass composting, and fertilizers). This subsystem contributes between 68% (FDP) and 98.5% (ME) of the impact in all the studied categories (Table 5). The specific analysis of the results is detailed in Section “Environmental Assessment Interpretation.”

**Table 6:** Environmental assessment of producing 1 kg of product.

	Infrastructure		Operation					Total
	Auxiliary	Wood		Emissions			Waste
	eq.	structure	Fertilizers	to water	Substrate	Water	biomass	
**CC**	9.1E-02	9.5E-03	3.9E-01	–	5.2E-03	3.9E-02	4.9E-03	5.5E-01
	**16.6%**	**1.7%**	**72.7%**		**1.0%**	**7.1%**	**0.9%**	
**TA**	4.1E-04	1.2E-04	2.9E-03	–	3.8E-05	1.9E-04	2.3E-05	3.7E-03
	**10.9%**	**3.2%**	**79.3%**		**1.0%**	**5.0%**	**0.6%**	
**FE**	2.8E-05	1.2E-06	8.4E-04	9.8E-04	5.9E-07	2.5E-05	1.6E-07	1.9E-03
	**1.5%**	**0.1%**	**44.8%**	**52.3%**	**<0.1%**	**1.3%**	**<0.1%**	
**ME**	7.1E-05	6.7E-06	2.3E-04	4.8E-03	2.4E-06	9.2E-06	1.3E-06	5.1E-03
	**1.4%**	**0.1%**	**4.4%**	**93.9%**	**<0.1%**	**0.2%**	**<0.1%**	
**ET**	3.5E-03	7.5E-04	6.1E-02	3.4E-03	4.9E-05	1.7E-03	1.2E-05	7.0E-02
	**5.0%**	**1.1%**	**86.6%**	**4.8%**	**0.1%**	**2.4%**	**<0.1%**	
**FDP**	3.8E-02	2.9E-03	7.4E-02	-	2.9E-03	1.1E-02	1.6E-03	1.3E-01
	**29.3%**	**2.3%**	**56.6%**		**2.2%**	**8.4%**	**1.2%**	

*Units: kg CO2 eq (CC, climate change), kg SO2 eq (TA, terrestrial acidification), kg P eq (FE, freshwater eutrophication), kg N eq (ME, marine eutrophication), kg oil eq (FD, fossil depletion), and kg 1-4DB eq (ET, ecotoxicity). Percentages express the relative contributions in each specific impact category*.

## Discussion

In this section, the production results are discussed and compared with the consumption data of the region. Finally, the environmental results shown in the results section are interpreted and discussed.

### Food Production Assessment

It is remarkable that an open-air system (present study) can obtain similar or higher productivity values than industrial or research greenhouses and conventional agriculture. This might be due to the standard seasons to which greenhouse production and conventional agriculture are bound. For instance, the typical season for eggplant is from May to September (120 days) (Aktas et al., 2013). Then, the crop is uprooted, and the next crop is planted following a monoculture production system. In contrast, the present study did not adhere to the conventional production seasons but rather extended them following the owner’s criteria and local weather conditions. Therefore, continuing with the previous example, in the study system, due to the favorable weather conditions (mainly due to the high minimum temperature), the eggplant crop was left until March (310 days). Consequently, the production season was extended, resulting in a higher final production.

These favorable local weather conditions could be affected by the urban heat island effect, which, compared with the outskirts of the city of Barcelona, can increase temperatures from 3 to 8°C (Ajuntament de Barcelona, 2018b). According to Mimet et al. (2009), the seasonal variations that urban crops undergo can be explained by the microclimates created in city centers, which, compared to rural areas, cause the earlier onset of the flowering phase. This alteration in the phenology of the plants leads to these extended seasons of production. Moreover, the heat island effect could offer new opportunities for cultivating vegetables that would not grow in the surrounding rural areas (Waﬄe et al., 2017). These crops could find sufficient climatic difference in cities to successfully grow during the winter seasons. This would be the case for tomato, eggplant and pepper, which have added value in winter considering that winter is not their typical season in the Mediterranean area. In winter, these crops are more difficult to find in markets, more expensive and usually imported, increasing their environmental impact.

The previous findings highlight the importance of garden management and the personal criteria of the owner, whose decisions affect the final production. Garden management can, for example, concern the water use efficiency of the system considering that water management plays a major role in soilless system performance compared to soil-based systems. In addition, water can be one of the elements with higher economic costs in UA systems (Sanyé-Mengual et al., 2015). Compared to other studies, such as Sanjuan-Delmás et al. (2018), the water use of the present system (1350.5 L/m^2^/year) is relatively high This fact has multiple explanations. First, it can be explained by the use of a single water circuit that irrigates the whole garden. The water flow was adjusted to the fruit crop water requirements, which turned out to be higher than the requirements of the leaf crops, resulting in a higher water consumption than that in monoculture systems where the water flow is adjusted to a crop-specific water requirement. Second, because the leachates were not quantified, there was no possibility that the garden owner could adjust the irrigation based on the drainage proportion. Moreover, the use of an open water irrigation system implies that all the irrigation water is consequently new water entering the system without any recirculation, which would immediately have an impact on new water consumption.

The selection of crops in this kind of garden is completely subjective and based on garden owner perception. For example, if the minimum temperature values are reached when a specific crop is growing and the crop does not succeed, the garden owner’s perception of the performance of this species could be highly altered, and as a consequence, the owner does not plant this crop again. Moreover, considering that crop selection is exclusively made by the garden owner, the crop variety and number of plants are not the same year after year. This fact makes data analysis more complex. In this sense, we suggest that in further research, an accurate analysis should be performed focusing on crop performance, especially in crops that can be grown in the off-season.

### Food Consumption

Table 3 shows the agronomic data for the products that were produced in the studied system and commonly consumed by Catalan citizens (market basket) according to official Catalan Administration sources (Gencat, 2016). They are ranked in descending order by the citizen consumption rates in kg per year. As shown in Table 3, only 2 products were consumed and not produced in the present system and were therefore missing from the list (position 2 for onion and 6 for carrot).

There were many other crops produced in the garden, such as cauliflower, broccoli, celery, etc., though consumption data were not available for these crops in the administration databases. The omission of these crops could be interpreted as their consumption being insignificant compared with other products. Nevertheless, the productivity of some of these crops in the present study was remarkable. Taken together, these results suggest that the crop inclusion criteria were based on the owner’s preference for certain vegetables or the preference for a mostly vegetarian diet, which would include a greater diversity and quantity of vegetables than those required by an average citizen. It could also be said that by managing a home garden, increased vegetable intake is almost inevitable, and thus, the average market basket does not fit the purpose anymore. This diet change based on higher vegetable intake represents a healthier diet, decreasing the chance of suffering diet-related chronic diseases (World Health Organization [WHO], 2015).

However, an important portion of the harvest was shared with other people. Having a vegetable surplus in a home garden can lead to the reinforcement of social relations by exchanging or giving the excess amounts to others. These practices enhance the creation and maintenance of social relations along with social cohesion and inclusion. Moreover, they can create networks of knowledge transmission and turn home gardens into agronomic learning places (Calvet-Mir et al., 2012).

The average fruit and vegetable consumption in Catalonia for 2015 and 2016 was 160 kg per person per year, excluding potatoes, which is more than the dietary intake recommended by the World Health Organization (World Health Organization [WHO], 2015). Nevertheless, of that amount, 104 kg were fruits, leaving a vegetable intake of only 56 kg per person per year. Those 56 kg were formed by 14 different vegetables, of which 9 have been grown in the studied system (Table 3). Based on the vegetable market basket and the production data, the area needed to fulfill the vegetable demand was calculated for 6 out of 9 products (the data were insufficient for the other crops). The results show that considering a density of 4 crops per square meter, 8.2 m^2^ would be necessary to satisfy 62% (by weight) of the vegetal demand for one person.

However, the average productivity of the present system was 10.6 kg/m^2^, from which it can be deduced that 5.3 m^2^ are needed per person to fulfill the vegetable demand, though this area does not include all the vegetables of the market basket. In this sense, improvements can be made to adapt the production to the consumption rates. For instance, more importance should be given to zucchini, beans and cucumber and less to eggplant and chard, whose production far exceeded the consumption rates. It should also be considered that diversifying the quantity of crops is necessary for avoiding the scenario in which only 5 products represent 78% of the total production, as happened in the present study.

The 5 crops present in the market basket that were not cultivated in the present study are onion, carrot, mushrooms, asparagus and garlic. There are no previous data on the feasibility of soilless cultivation in Mediterranean areas for these crops (Savvas et al., 2013). Similarly, fruit cultivation in soilless systems has not been studied. In the present system, the only fruits that were grown were melon, watermelon and strawberries, which are in positions 5, 6, and 20, respectively (per weight), in the consumption ranking of Catalonia. However, these crops were not successful in the present system.

A final possible consideration would be to increase the intake of the more successful vegetables among the community near the garden. The market basket was taken only as a reference of the variety and the quantity of the average vegetable consumption, but other vegetable combinations (varieties and quantities) are possible and healthy as well. The implementation of home urban gardens might boost the consumption of less popular and self-produced vegetables while decreasing the quantity of other fruits that might be imported (such as banana), thus reducing the overall environmental impact and increasing self-sufficiency.

### Environmental Assessment Interpretation

**Figure 4:**
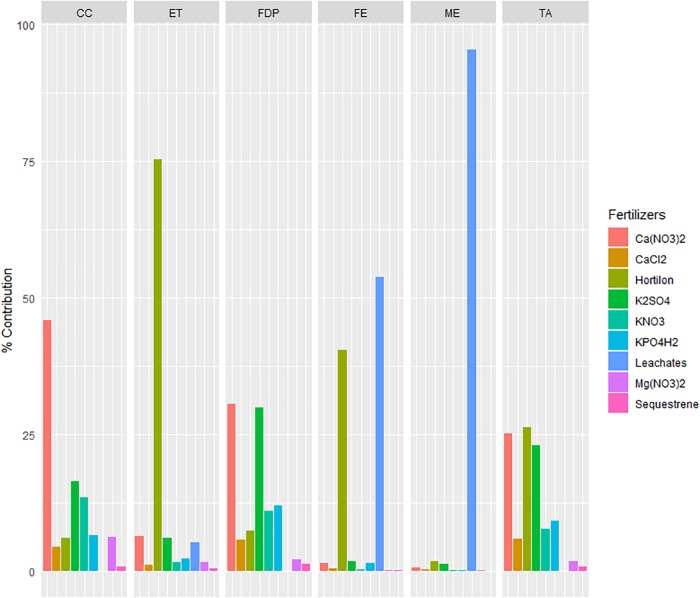
Relative impact of specific Fertilizers within the Fertilizer item in the life cycle inventory.

The greatest impact of the operation subsystem was mainly due to the fertilizers and their emissions, which were the elements with the greatest impact in all 6 of the impact categories studied. On the one hand, fertilizers accounted for between 56.6 and 86.6% of the impact in 4 out of the 6 impact categories (FDP, ET, TA, and CC). The relative impacts of the types of fertilizer are shown in Figure 4. Within fertilizers (Figure 3), Hortilon had the greatest impact for the ET and TA impact categories, accounting for 75.3 and 26.3% of the fertilizer impact, respectively. Moreover, it also exerted a great impact (0.15 kg of P eq. per year) in the FE category. However, the eutrophication impact categories were dominated by the drained leachates, both the FE (0.20 kg P eq. per year) and ME (0.96 kg of N eq. per year) categories. These impacts were highly significant due to the aforementioned assumption that most of the chemicals are not removed in the wastewater treatment plant and are considered direct emissions to the environment. Among the remaining fertilizers, only Ca(NO_3_)_2_ and K_2_SO_4_ had impacts above 20% of the fertilizer item impact. For instance, Ca(NO_3_)_2_ exerted the greatest impact in the CC (36.4 kg CO_2_ eq. per year; almost half of fertilizer impact) and FDP (4.48 kg oil eq. per year) categories. K_2_SO_4_ had similar impacts in this impact category (4.37 kg oil eq. per year).

Considering that the system is open (or linear), it is important to pay special attention to keeping the balance between not stressing or overwatering the plants so that the environmental impacts of the leachates are kept to a minimum and are not recirculated.

The network water used for the irrigation of the garden and the substrate had negligible impacts in most of the categories [between 8.4% (FDP) and 0.2% (ME) for water and between 2.2% (FDP) and 0.03% (FE) for the substrate].

The waste biomass treatment had the lowest impact, accounting for between 0.01 and 1.2% of the impact in the studied categories. Nevertheless, transport (municipal solid waste collection) far exceeded the impacts of the composting process, contributing over 97% of the impact in all the studied categories (Supplementary Information 1 Table A4). Similar results were found by Quirós et al. (2014).

The infrastructure impacts were mainly focused on the FDP (31.6%), CC (18.4%) and TA (14.1%) categories. Within the auxiliary equipment, the leachate tray (made of expandable polystyrene and HDPE) was the most impacting element, accounting for more than 40% of the impacts in 5 out of the 6 categories (CC, TA, ME, ET, and FDP).

For the wooden structure, the impacts accounted for between 3.2 and 0.1%, which is not very significant. Nevertheless, most of the structure was not directly used for the agricultural practice and had mainly decorative purposes. As a possible implementation, reducing the amount of wood to the minimum needed to support the stalking of the crops to minimize the impacts caused by this structure could be considered.

## Conclusion

This investigation showed the feasibility of a real polyculture soilless rooftop garden that achieved an average productivity of 10.6 kg/m^2^/year with a density of 4 plants/m^2^. The top 5 most productive crops were tomato, chard, lettuce, pepper and eggplant, accounting for 85.5% of the total production. Other crops, such as melon and green peas, were not as successful and had very low yields (<5 kg/year). Similarly, the water use was 3.7 L/m^2^/day, which is a relatively high compared to other studies.

Relative to food consumption, 9 out of the 14 vegetables that constitute the Catalan vegetable market basket were cultivated in the present study. In terms of the percentage covered, an 8.2 m^2^ soilless garden is sufficient for covering 62% of the Catalan vegetable market basket.

Regarding the environmental assessment, the urban home garden produced 5.9 kg of CO_2_ eq/m^2^/year. The majority of the impacts were caused during the operation phase (68.4 – 98.5% of impact among the 6 categories), particularly by the fertilizers in 4 out of the 6 analyzed impact categories (CC, TA, ET, FDP) and the resulting leachates for the other 2 categories (FE and ME). Compared to operation, infrastructure had the lowest impact, accounting for 1.5–31.6% of the impact.

The findings from this study make several contributions to the current literature on UA. Moreover, from the experience earned from this study, we detected which future research lines regarding this type of garden could follow. First, there should be cultivation trials of the crops included in the market basket but not assessed in the present study to assess their performance. Second, the fertilizer use (applying multiple crop-specific irrigation sectors) and leachate treatment (by promoting nutrient recirculation within the same system) should be optimized due to their great impacts. Finally, a rainwater harvesting system should be implemented considering that with the 2017 annual rainfall (442.1 mm) (IDESCAT, 2018), 29% of the water demand for the garden could be met.

## Author Contributions

AB acquired and gathered the data. Then, AB processed all the agronomic and environmental data to be included in the present article. AB also wrote the majority of the paper. MR-S assessed the environmental results and their interpretation as well wrote the article and discussed the results, especially the environmental assessment section. MR-S also integrated the majority of the revisions from the journal reviewers. ME-M assessed the agronomic results and their interpretation as well as wrote the article and discussed the results, especially the agronomic section. JR acquired and processed a portion of the data, assessed the global results as well as their interpretation and wrote the conclusions, and take-home messages. XG assessed the paper writing quality as well as the discussion section, especially the agronomic section.

## Conflict of Interest Statement

The authors declare that the research was conducted in the absence of any commercial or financial relationships that could be construed as a potential conflict of interest.
