# Supplementary material for: Agronomic and Environmental Assessment of a Polyculture Rooftop Soilless Urban Home Garden in a Mediterranean City
Source: Front Plant Sci. 2019 Mar 22;10:341. doi: 10.3389/fpls.2019.00341 (PMC6439533; doi:10.3389/fpls.2019.00341)
Supplement: Supplementary file 1 [file Table_1.docx]

**SUPPLEMENTARY INFORMATION**

**Agronomic and environmental assessment of a polyculture rooftop soilless urban home garden in a Mediterranean city**

Anna Boneta^a,1^, Martí Rufí-Salís^a,b,1^*, Mireia Ercilla-Montserrat^a^, Xavier Gabarrell^a,b^, Joan Rieradevall^a,b^

^a^Sostenipra Research Group 3.0 2017SGR 1683, Institute of Environmental Sciences and Technology (MDM-2015-0552), Z Building, Universitat Autònoma de Barcelona (UAB), Campus UAB, 08193 Bellaterra, Barcelona, Spain

^b^Department of Chemical, Biological and Environmental Engineering, Universitat Autònoma de Barcelona (UAB), Campus UAB, 08193 Bellaterra, Barcelona, Spain

**CORRESPONDENCE:**

Martí Rufí-Salís

[Marti.Rufi@uab.cat](mailto:Marti.Rufi@uab.cat)

^1^These authors contributed equally to the research

**Supplementary information 1: Life cycle inventory**

In this section, different tables gather all the data included in the life cycle inventory. The two firsts refer to the Infrastructure (table A.1) and Operation (table A.2) subsystems. Then, detailed inventories for Fertilisers (table A.3) and Waste biomass (table A.4) are also presented.

**Table A.1: Inventory of the infrastructure equipment**. (EoL for End-of-Life)

|  |  |  |  | **Quantity** | |
| --- | --- | --- | --- | --- | --- |
| **Element** | **Material** | **Lifespan** | **Units** | **garden**  **(10 years)** | **Per kg**  **(3 years)** |
| **Pipe** | PE | 10 | kg | 1.86 | 9.77E-04 |
| **Tubes** | PVC | 10 | kg | 0.63 | 3.33E-04 |
| **Drippers** | PVC | 10 | kg |  |  |
| **Leachate pipe** | LDPE | 10 | kg | 1.43 | 7.52E-04 |
| **Rain gutter** | PVC | 10 | kg | 1.69 | 8.90E-04 |
| **Leachate tray** | LDPE | 5 | kg | 2.95 | 3.10E-03 |
|  | EPS | 5 | kg | 22.68 | 2.39E-02 |
| **Joints** | PE | 10 | kg | 0.06 | 3.33E-05 |
| **Joints** | PE | 10 | kg | 0.06 | 3.16E-05 |
| **Support for stalking** | Aluminium | 10 | kg | 0.80 | 4.21E-04 |
| **Rope** | PP | 5 | kg | 0.45 | 4.79E-04 |
| **Flow meter** | HDPE | 10 | kg | 0.30 | 1.58E-04 |
|  | Cast iron | 10 | kg | 4.80 | 2.53E-03 |
| **Digital timer** | HDPE | 10 | kg | 0.50 | 2.63E-04 |
|  | Electronics | 10 | kg | 0.03 | 1.42E-05 |
| **Nutrient tank** | PE | 10 | kg | 8.10 | 4.27E-03 |
| **Dosatron** | PP | 10 | kg | 1.60 | 8.43E-04 |
| **Wood strucuture** | Wood | 10 | kg | 13.07 | 6.88E-03 |
| **PROCESSES** | | | | | |
| **Cast iron** | Metal working manufacturing | 10 | kg | 4.80 | 2.53E-03 |
| **HDPE** | Injection moulding | 10 | kg | 0.80 | 4.21E-04 |
| **PE** |  | 10 | kg | 10.08 | 5.31E-03 |
| **PP** |  | 10 | kg | 1.60 | 8.43E-04 |
|  |  | 5 | kg | 0.45 | 4.79E-04 |
| **PVC** | Extrusion, plastic pipes | 10 | kg | 2.32 | 1.22E-03 |
| **LDPE** |  | 5 | kg | 2.95 | 3.10E-03 |
| **LDPE** | Extrusion, plastic pipes | 10 | kg | 1.43 | 7.52E-04 |
| **EPS** | Injection moulding | 5 | kg | 22.68 | 2.39E-02 |
| **Transport** | Light commercial vehicle | 10 | tkm | 2.14 | 1.61E-03 |
| **Transport (EoL - recycling plant)** | Municipal waste collection, lorry | 10 | tkm | 0.30 | 1.52E-04 |
| **Transport (EoL - landfill)** |  | 10 | tkm | 1.56E-03 | 2.41E-04 |
| **Transport (EoL - green point)** | Van | 10 | tkm | 2.61E-02 | 1.38E-05 |
| **Transport (EoL - treatment wood str.)** | Municipal waste collection, lorry | 10 | tkm | 0.31 | 1.65E-04 |
| **Transport (EoL - gasification plant)** | Regional Lorry | 10 | tkm | 0.48 | 2.51E-04 |
| **EoL (aux. Eq.)** | Recycling plant | 10 | kg | 25.26 | 0.02 |
| **EoL** (leachate tray) | Landfill | 10 | kg | 22.68 | 2.39E-02 |

**Table A.2: Inventory of the operation and maintenance life cycle phases.**

| **Element** | **Material** | **Units** | **Quantity per kg for 3 years** |
| --- | --- | --- | --- |
| **Water** |  | L | 129.40 |
| **Substrate** | Perlite | kg | 5.06E-02 |
|  | HDPE | kg | 1.34E-03 |
| **Fertilisers** | KPO4H2 | kg | 1.76E-02 |
|  | KNO3 | kg | 2.09E-02 |
|  | K2SO4 | kg | 4.50E-02 |
|  | Ca(NO3)2 | kg | 5.53E-02 |
|  | Mg (NO3)2 | kg | 2.20E-02 |
|  | Ca (Cl)2.2H2O | kg | 1.90E-02 |
|  | Sequestone | kg | 1.29E-02 |
|  | Hortillon | kg | 1.29E-02 |
| **PROCESSES** | | | |
| **HDPE** | Extrusion. plastic film | kg | 1.34E-03 |
| **Leachates** | Cl^-^ | kg | 1.55E-04 |
|  | NO^-^_3_ | kg | 2.19E-02 |
|  | PO^3-^_4_ | kg | 3.65E-03 |
|  | SO^2-^_4_ | kg | 7.45E-03 |
|  | K^+^ | kg | 9.99E-03 |
|  | Mg^2+^ | kg | 2.68E-05 |
|  | Ca^2+^ | kg | 1.54E-03 |
| **Transport** (fertilisers) | Transport. van | tkm | 6.79E-03 |
| **Transport** (perlite) | Transport. lorry | tkm | 4.15E-02 |
| **Transport** (end of life - landfill) | Light commercial vehicle | tkm | 2.18 |
| **Transport** (end of life - OMSW treatment plant) | Light commercial vehicle | tkm | 4.18E-03 |

**Table A.3: Biomass detailed impact inventory.**

Units: kg CO2 eq (CC, Climate Change), kg SO2 eq (TA, Terrestrial acidification), kg P eq (FE, Freshwater Eutrophication), kg N eq (ME, Marine Eutrophication), kg oil eq (FD, Fossil Depletion), kg 1-4DB eq (ET, Ecotoxicity

|  | **CC** | **TA** | **FE** | **ME** | **ET** | **FDP** |
| --- | --- | --- | --- | --- | --- | --- |
| Transport | 4.77E-03  98.0% | 2.20E-05  96.5% | 1.58E-07  98.7% | 1.26E-06  97.1% | 1.12E-05  97.3% | 1.55E-03  99.8% |
| Biowaste composting | 9.73E-05  2.0% | 7.94E-07  3.5% | 2.12E-09  1.3% | 3.71E-08  2.9% | 3.13E-07  2.7% | 3.45E-06  0.2% |
| Total | 4.87E-03 | 2.28E-05 | 1.60E-07 | 1.29E-06 | 1.16E-05 | 1.55E-03 |

Supplementary information 2: agronomic data

**Table B.1. Summarized agronomic and consumption data for the market basket products.**

Data for the kg/person. Data for consumption ranking (Gencat, 2016).

| Ranking position | Products | Year | Production  **kg/year** | Consumption  **kg/person/year** | nº people's covered demand | m^2^ to cover 1 person’s demand |
| --- | --- | --- | --- | --- | --- | --- |
| 1 | **TOMATO** | 2015 | 54.5 | 16.5 | 3.3 | 2.8 |
|  |  | 2016 | 29.8 |  | 1.8 |  |
|  |  | 2017 | 35.6 |  | 2.2 |  |
| 3 | **LETTUCE** | 2015 | 22.3 | 5.1 | 4.4 | 3.5 |
|  |  | 2016 | 27.4 |  | 5.4 |  |
|  |  | 2017 | 19.6 |  | 3.9 |  |
| 4 | **PEPPER** | 2015 | 30.4 | 4.7 | 6.4 | 0.4 |
|  |  | 2016 | 25.2 |  | 5.3 |  |
|  |  | 2017 | 30.3 |  | 6.4 |  |
| 5 | **ZUCCHINI** | 2015 | - | 4.2 | - | 1.1 |
|  |  | 2016 | - |  | - |  |
|  |  | 2017 | 1.0 |  | 0.2 |  |
| 7 | **BEAN** | 2015 | - | 3.0 | - | - |
|  |  | 2016 | 4.5 |  | 1.5 |  |
|  |  | 2017 | 5.5 |  | 1.8 |  |
| 8 | **CUCUMBER** | 2015 | 1.0 | 2.4 | 0.4 | - |
|  |  | 2016 | 1.0 |  | 0.4 |  |
|  |  | 2017 | 2.5 |  | 1.1 |  |
| 9 | **EGGPLANT** | 2015 | 15.3 | 2.1 | 7.2 | 0.1 |
|  |  | 2016 | 29.7 |  | 14.0 |  |
|  |  | 2017 | 27.6 |  | 13.0 |  |
| 10 | **CABBAGE** | 2015 | 15.7 | 1.7 | 9.2 | 0.4 |
|  |  | 2016 | - |  | - |  |
|  |  | 2017 | - |  | - |  |
| 11 | **CHARD** | 2015 | 33.5 | 1.5 | 21.9 | - |
|  |  | 2016 | 14.6 |  | 9.5 |  |
|  |  | 2017 | 32.9 |  | 21.5 |  |
